# Rethinking Tuberculosis Treatment Abandonment in Latin America: A Biopsychosocial Perspective and an Integrated Model for Continuity of Care

**DOI:** 10.3390/diseases14080267

**Published:** 2026-07-24

**Authors:** Ariel Torres, Paloma González, Martha Fors, Gisselle Trujillo

**Affiliations:** 1 Facultad de Ciencias de la Salud y Bienestar Humano, Universidad Tecnológica Indoamérica, Quito 170103, Ecuador; arieltorres@uti.edu.ec (A.T.); gisselletrujillo@uti.edu.ec (G.T.); 2 Biblioteca, Universidad de Las Américas, Quito 170125, Ecuador; 3 One Health Research Group, Facultad de Ciencias de la Salud, Universidad de Las Américas, Quito 170125, Ecuador; martha.fors@udla.edu.ec

**Keywords:** tuberculosis, treatment abandonment, social vulnerability, retention in care, Latin America

## Abstract

**Background:** Tuberculosis remains a major public health challenge in Latin America, where social inequalities, fragmented health systems, and barriers to care continue to undermine disease control. Although effective treatment is available, treatment abandonment and loss to follow-up remain persistent obstacles to successful outcomes. **Methods**: A structured critical analysis of the contemporary literature on tuberculosis treatment abandonment and retention in care was conducted using a narrative evidence synthesis approach. Forty scientific documents published between 2018 and 2026 were included. Of these, 23 Latin American studies informed the results synthesis, while 13 international studies supported the comparative critical reflection. **Results:** Findings were organised into five domains: social vulnerability, clinical complexity, health system barriers, priority populations, and protective factors and innovation. The evidence indicates that treatment abandonment is not an isolated individual behaviour, but a phenomenon associated with the convergence of social, clinical, and institutional determinants across the care trajectory. Poverty, migration, drug resistance, incarceration, weak follow-up systems, and fragmented care were repeatedly associated with discontinuity, whereas social support, active follow-up, digital tools, and economic measures were linked to improved adherence and retention in care. **Conclusions:** From a biopsychosocial perspective, treatment abandonment should be understood as a systemic and preventable failure across the tuberculosis care continuum. The proposed Latin American Integrated Model for Retention in Tuberculosis Care (MILERTB) offers an anticipatory, equity-oriented, and person-centred framework to strengthen retention in care and guide future implementation research.

## 1. Introduction

Tuberculosis (TB) remains one of the leading infectious diseases worldwide and continues to be highly relevant in Latin America, where social inequalities, barriers to healthcare access, and fragmented health systems persist. Although TB is preventable and curable, treatment abandonment remains a major obstacle to its control, as it is associated with therapeutic failure, relapse, sustained transmission, and increased costs for public health programmes [1].

In the Region of the Americas, the burden of TB is concentrated among vulnerable populations, including people living in poverty, prisoners, migrants, Indigenous communities, and residents of deprived urban areas. In these contexts, maintaining prolonged treatment regimens is often hindered by economic hardship, the need for daily work, transport costs, social stigma, and limited continuity of care [2,3].

Treatment abandonment is further exacerbated by its relationship with drug resistance. Irregular or premature interruption favours resistant mutations, increasing the risk of rifampicin-resistant and multidrug-resistant tuberculosis (MDR-TB), and, in more advanced forms, pre-XDR/XDR-TB, where treatment becomes longer, more toxic, and less effective [1]. In Latin America, diagnostic limitations, unequal access to rapid testing, and variable availability of second-line drugs intensify this challenge, positioning treatment abandonment as both a clinical and structural issue [2].

From this perspective, treatment abandonment should not be interpreted solely as an individual behaviour, but as the result of cumulative system failures, including insufficient follow-up, rigid service delivery, limited health education, and inadequate psychosocial support. Consequently, international guidelines increasingly promote person-centred approaches, social protection measures, and supported adherence over punitive models [4].

In this context, Latin America must move from reactive responses to preventive strategies based on early risk identification. Targeting patients with greater clinical and social vulnerability from treatment initiation would enable interventions such as community follow-up, financial support, digital monitoring, and interdisciplinary care. Despite the growing body of research, evidence remains fragmented across countries and perspectives, with limited efforts to integrate social, clinical, and health system determinants within a unified regional framework while incorporating global insights. Consequently, there remains a lack of integrative approaches that systematically connect these determinants across the tuberculosis care continuum in Latin America.

Previous experiences from different world regions have highlighted that tuberculosis treatment adherence is influenced by a complex interaction of social, economic, behavioural, and health system determinants rather than by clinical factors alone. International reports and guidelines increasingly advocate person-centred approaches, social protection measures, patient support interventions, and strengthened continuity of care as essential components of tuberculosis control strategies [1,3]. Similarly, evidence from settings outside Latin America has shown that social vulnerability, poverty, and structural barriers may substantially affect treatment continuity and therapeutic outcomes, reinforcing the need for comprehensive and integrated approaches to retention in care [4]. These observations suggest that successful tuberculosis control requires health systems capable of addressing both the biomedical and the broader social determinants that influence long-term adherence [1,2,3,4].

The novelty of this study lies in its integrative perspective. Rather than examining isolated determinants of treatment abandonment, it synthesises evidence across social, clinical, and health system domains and connects these dimensions within a unified biopsychosocial framework. Furthermore, by incorporating both Latin American and international evidence, the study proposes the Latin American Integrated Model for Retention in Tuberculosis Care (MILERTB), a conceptual framework intended to support future programme design, implementation research, and continuity-of-care strategies in the region.

Accordingly, this study aimed to critically analyse the determinants of tuberculosis treatment abandonment in Latin America, compare regional findings with evidence from other world regions, and propose an integrated biopsychosocial framework to strengthen retention in care.

## 2. Materials and Methods

This study was conducted as a structured critical analysis of the contemporary scientific literature addressing tuberculosis treatment abandonment and retention in care, with a particular focus on Latin America. Using a narrative evidence synthesis approach, the study aimed to identify, integrate, and critically interpret recurring social, clinical, and health system determinants reported across diverse epidemiological contexts. The ultimate purpose of this analytical process was to develop an integrative biopsychosocial framework capable of supporting a more comprehensive understanding of continuity of care and informing the formulation of the Latin American Integrated Model for Retention in Tuberculosis Care (MILERTB). This approach prioritised conceptual integration, thematic consistency, and explanatory capacity rather than quantitative aggregation of findings.

A documentary search was conducted in indexed databases (PubMed/MEDLINE, Scopus, and Google Scholar), using combinations of terms in Spanish and English related to tuberculosis, treatment abandonment, adherence, and continuity of care, prioritising studies published between 2018 and 2026. The search strategy combined terms such as “tuberculosis”, “treatment abandonment”, “loss to follow-up”, “adherence”, and “retention in care”, using Boolean operators (AND/OR), adapted to each database. For the purposes of this study, treatment abandonment and loss to follow-up (LTFU) were recognised as conceptually related but not necessarily identical phenomena across tuberculosis programmes and studies. However, both terms were considered indicators of discontinuity in care and were analysed jointly within the broader framework of treatment retention and continuity of care. An example of the search strategy used in PubMed was: (“tuberculosis” AND (“treatment abandonment” OR “loss to follow-up” OR “adherence” OR “retention in care”)). Equivalent adaptations were applied to the remaining databases according to their indexing systems and search functionalities.

The selection process was based on criteria of thematic relevance, methodological pertinence, and coherence with the study objectives. Inclusion criteria comprised studies addressing tuberculosis treatment abandonment, loss to follow-up, adherence, or continuity of care in human populations, with a particular emphasis on social, clinical, or health system determinants. Exclusion criteria included studies focused exclusively on microbiological or laboratory aspects without relevance to treatment continuity, as well as documents lacking sufficient analytical contribution to the study objectives. Study selection was conducted through iterative screening by all authors, based on thematic relevance, conceptual contribution, and alignment with the analytical framework. Disagreements regarding study inclusion were resolved through discussion and consensus.

From this strategy, an initial pool of studies was identified, from which, following a process of refinement based on relevance and conceptual quality, a final corpus of 40 documents was established. Of these, four were used as conceptual support in the Introduction, 23 studies from Latin America were included in the results synthesis, and 13 studies from other regions were incorporated into the comparative critical reflection. A detailed summary of the characteristics, study designs, populations, and principal findings of the 23 Latin American studies included in the primary evidence synthesis is provided in Supplementary Table S1. The distribution of references according to their role within the analytical synthesis is presented in Table 1.

The international studies were selected for their contribution to understanding global patterns of treatment adherence, retention in care, health information systems, social determinants, and innovative interventions aimed at reducing loss to follow-up across different epidemiological and healthcare contexts.

**Figure 1:**
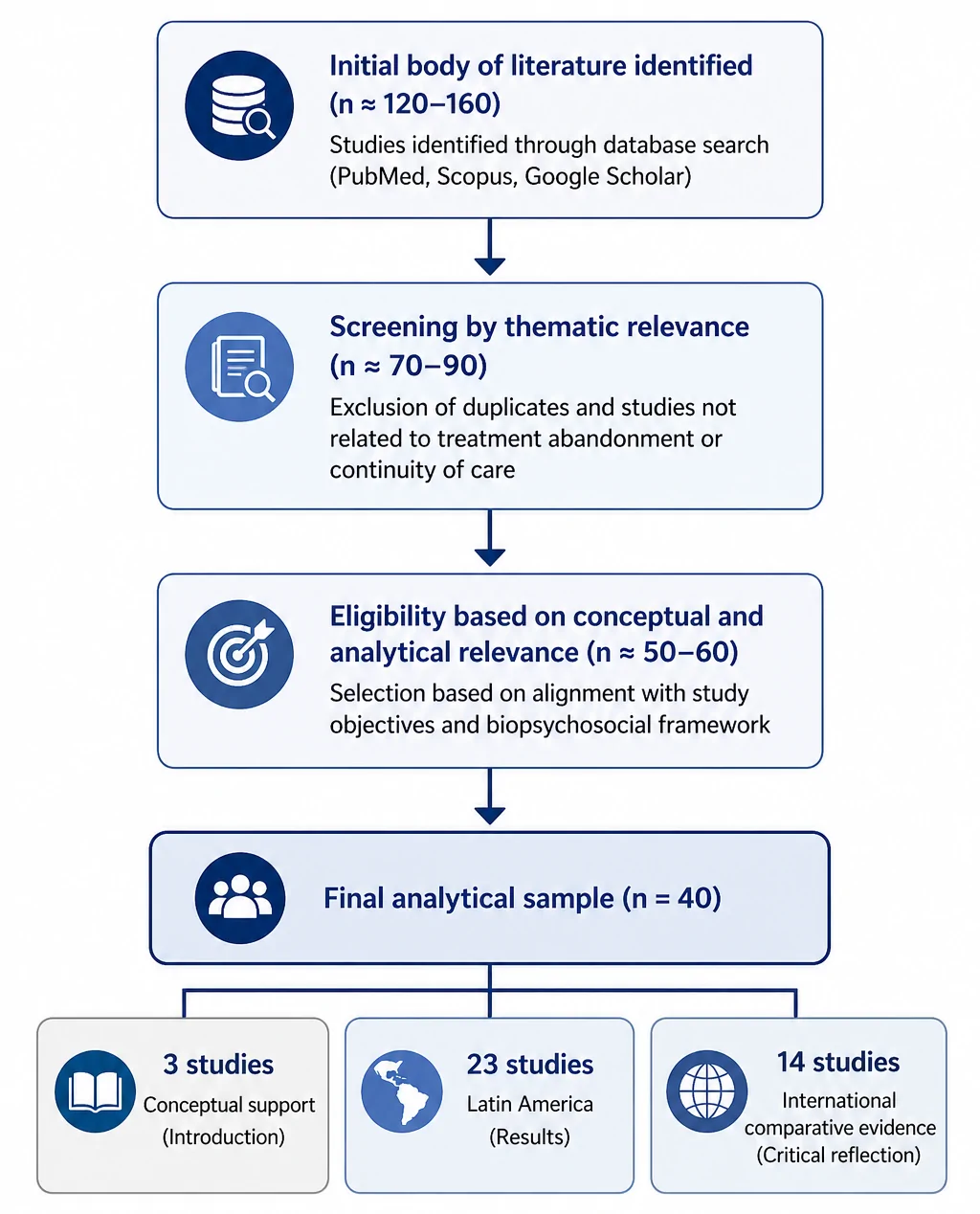
Narrative selection process of studies included in the critical synthesis. This figure illustrates the narrative selection process of the included studies, detailing the progression from the initial pool of identified records through screening and refinement stages to the final analytical sample of 40 documents. Although not following a formal PRISMA framework, it provides a transparent representation of the selection logic applied.

To ensure transparency in this selection process, a graphical representation (Figure 1) was developed to describe the progression from the initial pool of identified studies to the final analytical sample; this does not correspond to a PRISMA diagram but rather to a narrative depiction of the inclusion logic.

For each included study, information was extracted regarding the country of origin, study characteristics, population groups analysed, determinants associated with treatment abandonment or loss to follow-up, reported barriers to continuity of care, and interventions or protective factors related to treatment retention. The extracted information was organised into an analytical matrix to facilitate comparison, thematic integration, and critical interpretation across studies.

The information was analysed through a thematic narrative synthesis, organising the findings into five domains: social vulnerability, clinical complexity, health system barriers, priority populations, and protective factors and innovation, which allowed the identification of consistent patterns and relationships among determinants of treatment abandonment across the care trajectory. The five thematic domains were not predefined a priori but emerged through an iterative process of comparative analysis and thematic convergence. Categories were retained when similar determinants, barriers, or facilitating factors were consistently identified across multiple studies and contexts, allowing the organisation of recurring patterns into analytically coherent domains.

In addition to thematic organisation, the synthesis incorporated a comparative interpretative approach aimed at identifying recurrent determinants, areas of convergence across countries, and patterns consistently reported in different epidemiological and healthcare settings. The purpose was not to establish quantitative rankings of determinants, but to explore the consistency and contextual relevance of the factors associated with treatment abandonment and loss to follow-up.

As part of the analytical process, a country-level comparative matrix (Table 2) was developed, integrating the level of available evidence, the magnitude of the problem, and the degree of implementation of strategies. For this purpose, explicit qualitative criteria were defined: the level of evidence was classified as high, medium, low, or absent according to the number and consistency of identified studies; the magnitude of the problem was categorised as high, medium, low, or not evaluable based on the convergence of reported factors; and the level of implementation of strategies was defined as high, medium, low, or not documented, considering the presence and diversity of described interventions. These classifications were applied through an interpretative synthesis process, without predefined quantitative thresholds, prioritising conceptual consistency and comparability across heterogeneous contexts. The purpose of this matrix was not to establish quantitative rankings between countries, but to provide a structured comparative overview of the available evidence, the reported magnitude of treatment abandonment, and the extent of documented retention strategies across heterogeneous national contexts.

The category “not evaluable” was incorporated to avoid misinterpretation in contexts with absence of evidence, prioritising a reading of the table as a map of evidence rather than as a comparative ranking.

Although the classifications incorporated in the comparative matrix were based on explicit qualitative criteria, they inevitably involved a degree of interpretative judgement. The matrix should therefore be understood as an analytical tool designed to facilitate cross-country comparison of recurring patterns rather than as a definitive ranking system. Future studies employing formal consensus methods or quantitative indicators may further improve reproducibility and external validation of this framework.

**Figure 2:**
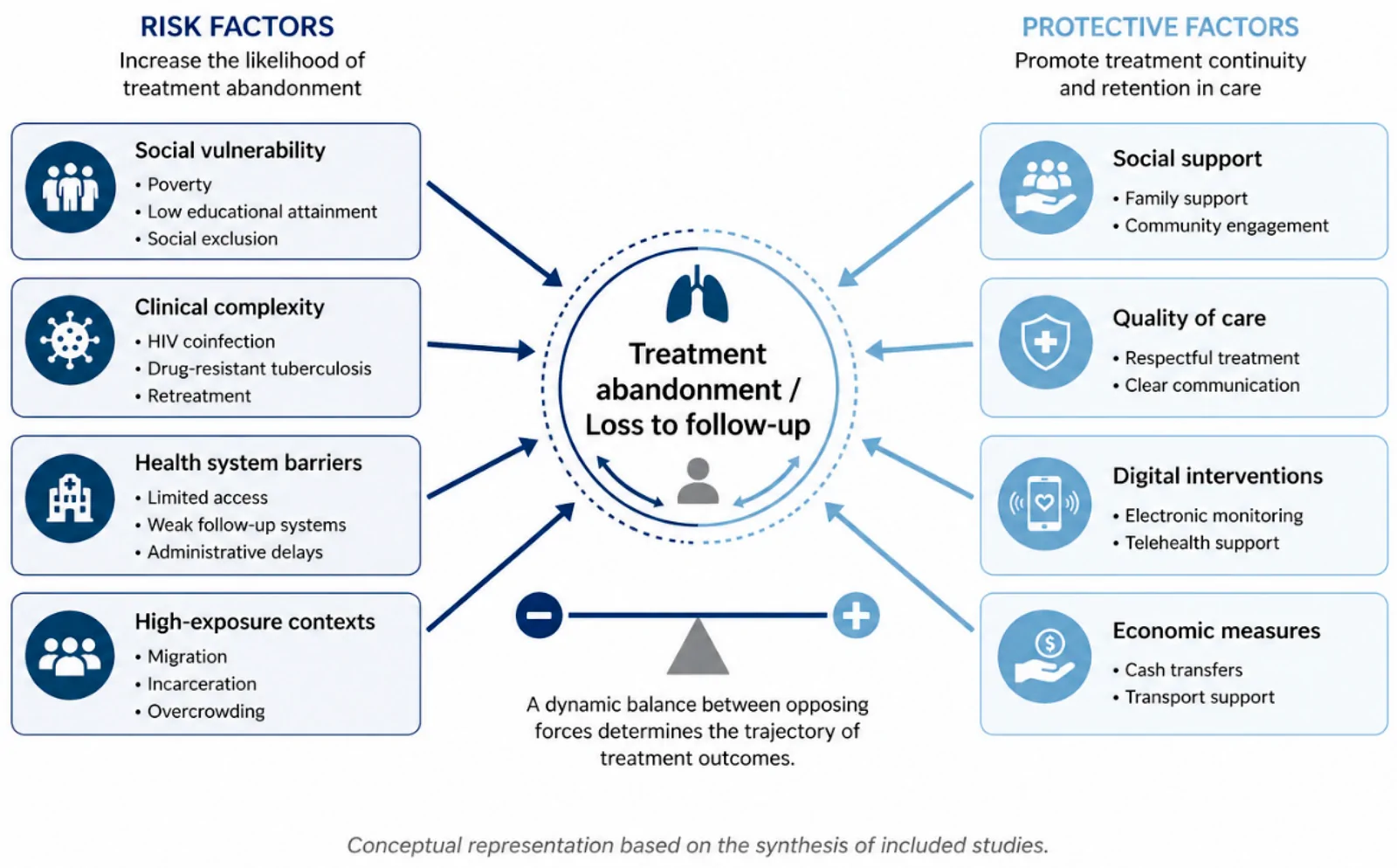
Conceptual scheme of interacting determinants of tuberculosis treatment abandonment and retention in care. This figure presents a conceptual representation of treatment abandonment as the result of the interaction between risk factors and protective elements across the care trajectory. Social vulnerability, clinical complexity, health system barriers, and high-exposure contexts are associated with an increased risk of loss to follow-up, whereas social support, quality of care, digital interventions, and economic measures act as stabilising factors that promote treatment continuity and retention in care.

Based on the integration of the findings, a conceptual scheme (Figure 2) was developed to provide a visual synthesis of the principal determinants associated with treatment abandonment and the protective factors linked to retention in care. The figure represents treatment abandonment as the result of the interaction between risk factors and protective elements, synthesising the convergence of social, clinical, and institutional determinants, as well as their relationship with strategies associated with retention in care, thereby facilitating interpretation of the relationships identified across the included studies. The balance represented in Figure 2 should be interpreted conceptually rather than quantitatively. Its purpose is to illustrate how the accumulation of risk factors may increase vulnerability to treatment discontinuity, whereas the presence of protective elements may strengthen retention in care. The figure does not propose a scoring system or predictive model, but rather a framework for understanding the dynamic interaction between determinants across the care trajectory.

**Figure 3:**
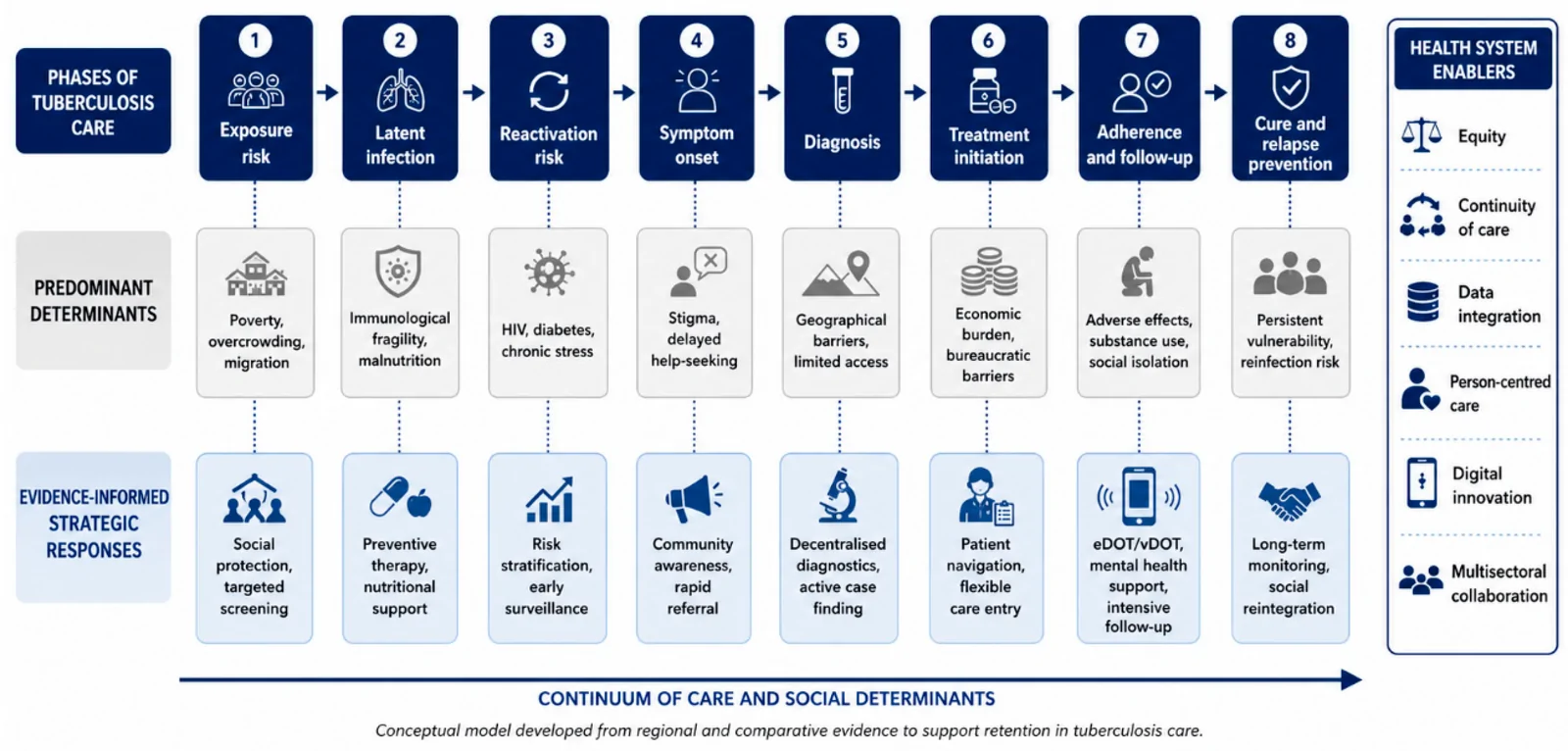
Latin American Integrated Model for Retention in Tuberculosis Care (MILERTB): biopsychosocial continuum, determinants, and strategic interventions. This figure presents the Latin American Integrated Model for Retention in Tuberculosis Care (MILERTB), illustrating a biopsychosocial continuum of care from exposure risk to cure and relapse prevention. The model integrates predominant determinants across each phase of the care trajectory with evidence-informed strategic responses, highlighting opportunities for early intervention and sustained retention in care. Health system enablers, including equity, continuity of care, data integration, person-centred care, digital innovation, and multisectoral collaboration, are depicted as cross-cutting components supporting the effective implementation of the model.

Additionally, and as a derivation of the comparative critical reflection, an integrative model named the Latin American Integrated Model for Retention in Tuberculosis Care (MILERTB) was formulated, presented in Figure 3, proposing an anticipatory and person-centred approach to prevent treatment abandonment across the entire continuum of care. This model was constructed through an interpretative synthesis process based on the articulation of regional and global evidence.

Finally, generative artificial intelligence tools were used exclusively to support the visual design of figures (Figure 1, Figure 2 and Figure 3), without involvement in the selection, analysis, or interpretation of the evidence. All analytical decisions were made by the authors, in accordance with principles of transparency and academic responsibility. As this study was based exclusively on publicly available secondary sources, ethical approval was not required, and scientific integrity, appropriate citation of sources, and responsible use of digital technologies were ensured throughout.

## 3. Results

### 3.1. Social Vulnerability, Poverty, and Structural Exclusion

Across the region, studies consistently report patterns linking social vulnerability to increased risk of treatment abandonment, with variations across countries reflecting contextual socioeconomic and health system differences.

The reviewed evidence consistently showed an association between social vulnerability and an increased risk of treatment abandonment, loss to follow-up (LTFU), or unfavourable tuberculosis outcomes. In Brazil, Lima et al., Ryuk et al., Lucena et al., and Mendonça et al. [5,6,7,8] identified a higher frequency of treatment interruption among individuals with low educational attainment, poverty, unemployment, homelessness, and substance use, as well as regional inequalities. Chenciner et al. [9] reported similar patterns among socially vulnerable adolescents and young adults.

In Mexico, Sánchez-Pérez et al. [10] documented poorer outcomes among patients experiencing poverty, living in dispersed rural areas, facing geographical barriers, and social exclusion in Chiapas. In Colombia, Varela et al. and Perlaza et al. [11,12] described an association between socioeconomic vulnerability, financial hardship, and a higher probability of unfavourable outcomes or treatment abandonment.

In Peru, Tapia-Tenorio et al., Chiang et al., and Brooks et al. [13,14,15] identified the influence of economic barriers, employment-related difficulties, family dependency, and logistical barriers in maintaining treatment continuity. In Paraguay, Escobar et al. [16] reported economic vulnerability and limited social support, while Medina et al. [17] highlighted the concentration of tuberculosis among people deprived of liberty and Indigenous populations. In Chile, Muñoz et al. [18] observed a higher tuberculosis burden in areas characterised by migration, overcrowding, and lower income levels.

### 3.2. Clinical Complexity and High-Risk Treatment History

In addition to social determinants, studies identified that clinical complexity and high-risk treatment history were associated with a greater probability of treatment abandonment, loss to follow-up, or unfavourable outcomes. In Brazil, Lima et al. and Ryuk et al. [5,6] reported HIV coinfection, comorbidities, and complex clinical histories as key predictors of treatment interruption. Complementarily, Barreto-Duarte et al. [19] documented poorer outcomes among patients undergoing retreatment, with higher frequencies of recurrent abandonment, treatment failure, and new episodes of loss to follow-up.

In Colombia, Varela et al. [11] described increased vulnerability among previously treated patients, particularly in the presence of relapse, retreatment, coinfections, and greater clinical burden. In Mexico, Flores-Treviño et al. [20] identified previous tuberculosis episodes, prior exposure to anti-tuberculosis drugs, and diagnostic delays as relevant factors in drug-resistant tuberculosis.

Ecuador, Tatés-Ortega et al. [21] documented loss to follow-up among patients with rifampicin-resistant or multidrug-resistant tuberculosis undergoing prolonged treatment regimens, high medication burden, and adverse effects. Similarly, Romero-Sandoval et al. [22] reported complex treatment trajectories and continuity challenges in prison populations with high epidemiological vulnerability.

### 3.3. Health System Barriers and Programmatic Weakness

In addition to social and clinical factors, several studies reported barriers related to the functioning of health services and the operational capacity of tuberculosis programmes. In Brazil, Ryuk et al. and Mendonça et al. [6,8] identified regional differences and territorial heterogeneity in treatment outcomes, suggesting variations in access, follow-up, and programme performance. Lucena et al. [7] also described weak follow-up systems and regional inequalities among factors associated with treatment abandonment.

In Colombia, Perlaza et al. [12] documented poor continuity of care, administrative delays, weak programme follow-up, and limited health education within the public health system. In Mexico, Sánchez-Pérez et al. [10] reported reduced continuity of follow-up and access barriers in dispersed rural areas.

In Peru, Chiang et al. [14] described rigid requirements of directly observed treatment, communication difficulties between users and health services, and limited adaptation of the system to adolescents’ needs. Brooks et al. [15] identified institutional limitations in actively retaining exposed children and their caregivers throughout the care process. In Ecuador, Romero-Sandoval et al. [22] reported operational barriers and insufficient health system coordination within prison settings.

### 3.4. Priority Populations and High-Vulnerability Contexts

The reviewed studies also identified specific population groups and contexts with a higher risk of treatment abandonment, loss to follow-up, or unfavourable outcomes. In Brazil, Chenciner et al. [9] reported a substantial proportion of unsuccessful outcomes among adolescents and young adults, associated with social instability, mobility, and economic vulnerability. Ryuk et al. [7] also noted a higher concentration of adverse outcomes in socially vulnerable groups.

In Peru, Chiang et al. [14] described specific barriers among adolescents, including treatment fatigue, educational or occupational constraints, and dependence on family members to attend follow-up visits. Brooks et al. [15] documented loss to follow-up among child contacts of tuberculosis cases across different stages of evaluation and treatment.

In Ecuador, Romero-Sandoval et al. [22] identified complex treatment trajectories among people deprived of liberty in the largest male prison in the country. In Paraguay, Medina et al. [17] reported a disproportionate concentration of tuberculosis among incarcerated individuals and Indigenous populations. In Chile, Muñoz et al. [18] observed a higher disease burden in areas with a high concentration of migrant populations.

### 3.5. Protective Factors and Innovation to Improve Treatment Adherence

Finally, several studies identified factors associated with improved treatment continuity and strategies aimed at strengthening adherence. In Peru, Dilas et al. [23] reported that perceived social support and quality of care were associated with higher treatment adherence, while health education provided by nursing staff acted as a favourable mediating factor. Chiang et al. [14] described facilitators such as family support, respectful treatment by healthcare personnel, clear information, and continuous follow-up. Acosta et al. [24] found that an electronic medication monitoring system improved treatment regularity and enabled early detection of missed doses.

In Brazil, Rodrigues et al. [25] developed predictive models using machine learning to identify patients at higher risk of loss to follow-up from early stages of treatment. Jesus et al. [26] reported that conditional cash transfer programmes were associated with reductions in tuberculosis burden and mortality in vulnerable populations. In Argentina, Discacciati [27] synthesised contemporary strategies to improve treatment adherence in outpatient settings, including health education, community-based support, digital reminders, psychological support, and reduction of economic or logistical barriers.

To comparatively synthesise the available evidence across the region, Table 2 was developed at the country level, integrating the level of evidence, the magnitude of the problem, and the degree of implementation of strategies aimed at improving adherence and retention in care. This approach allows visualisation not only of the burden of treatment abandonment or loss to follow-up, but also of the programmatic response capacity in each context. It is important to note that the interpretation of these levels does not imply a hierarchical comparison between countries, but rather an approximation to the state of the evidence and the articulation of reported interventions, incorporating the category of “not evaluable” in settings where studies are absent. The classification was based on qualitative synthesis rather than quantitative scoring, aiming to provide an interpretative mapping of the available evidence rather than a hierarchical comparison between countries.

In Figure 2, a conceptual scheme is presented that organises the findings around treatment abandonment, understood as the result of the interaction between risk factors and protective elements. Social vulnerability, clinical complexity, health system barriers, and high-exposure contexts are associated with loss to follow-up, whereas social support, quality of care, digital interventions, and economic measures act as factors that favour treatment continuity.

Across the included studies, treatment abandonment and loss to follow-up were reported in association with social vulnerability, clinical complexity, and health system barriers across different stages of the care trajectory. Additionally, interventions such as social support, active follow-up, digital strategies, and economic measures were reported in association with improved adherence and retention in care.

## 4. Discussion

Regional findings indicate that tuberculosis treatment abandonment in Latin America extends beyond the notion of individual non-compliance. Rather, it arises from the interaction between social vulnerability, clinical complexity, and institutional limitations. The simultaneous presence of poverty, low educational attainment, substance use, HIV coinfection, retreatment, incarceration, migration, and territorial barriers shapes fragile care trajectories, in which loss to follow-up may begin even before the formal initiation of treatment, during delayed diagnosis, limited health education, or the absence of social support.

International comparisons reinforce this interpretation. In Europe, Cotugno et al. [28] documented poorer treatment outcomes among migrant populations, associated with mobility, language barriers, and legal insecurity. This suggests that even in health systems with greater capacity, treatment continuity deteriorates when services fail to respond to mobile or socially vulnerable populations. In Latin America, where recent migration, labour informality, and territorial exclusion are common, this phenomenon may be amplified.

Another relevant aspect is the quality of health information systems. Kim et al. [29], in South Korea, identified “hidden loss to follow-up”, demonstrating that part of treatment abandonment may remain unrecorded due to fragmented data systems or administrative closures. This observation is particularly pertinent to Latin America, where interoperability between primary care, hospitals, prisons, and specialised programmes remains limited. In this context, some favourable indicators may overestimate the true success of treatment.

Evidence from Indonesia and Canada also shows that drug-resistant tuberculosis carries a higher risk of treatment interruption due to prolonged regimens, toxicity, treatment fatigue, and the need for specialised follow-up [30,31,32]. This pattern aligns with Latin American studies that identify retreatment, previous abandonment, and drug resistance as scenarios of heightened vulnerability. In such cases, treatment adherence depends less on individual will and more on the system’s capacity to sustain intensive clinical support.

From this perspective, the central question should not be solely why patients abandon treatment, but rather which conditions hinder the system’s ability to retain them in care. When treatment competes with hunger, costly transport, precarious employment, or stigma, treatment continuity becomes structurally constrained [4]. In line with this, Alipanah et al. [33] found that the most effective interventions combine material support, education, psychosocial accompaniment, and the reduction of practical barriers, rather than relying on a single measure. This pattern is consistent with evidence from Central Asia, where social determinants such as poverty, inequality, and structural vulnerability have been shown to significantly influence tuberculosis incidence and outcomes [34].

Digital technologies represent a relevant, though not universal, opportunity to enhance treatment adherence. Jerene et al. [35], through four pragmatic cluster-randomised trials, demonstrated that digital adherence technologies can improve treatment outcomes among people with drug-susceptible tuberculosis when appropriately integrated into routine care. Similarly, Santosa et al. [36] demonstrated that electronic tools can improve adherence when effectively integrated with human support, while Burzynski et al. and Perry et al. [37,38] showed that electronic and video-based observation can maintain therapeutic supervision with greater flexibility. However, in Latin America, their implementation requires careful consideration of structural constraints, including limited connectivity, variable digital literacy, and unequal access to devices. Without an equity-oriented approach, these innovations risk benefiting only certain groups and may ultimately reinforce existing health disparities.

Community-based strategies appear particularly relevant for the region. Kedthongma et al. [39] confirmed that locally based support interventions enhance treatment adherence and completion, while evidence from Latin America highlights the importance of social support and community-linked care in improving treatment continuity [24]. In Latin America, where marked territorial inequalities persist, community-based models may bring care closer to rural, peripheral, and socially excluded populations more effectively than highly centralised approaches.

Social protection constitutes another key component. Rubinstein and Blumenfeld [40] noted that cash transfer programmes can improve treatment continuity, although they remain insufficient if not accompanied by accessible services and quality care. This observation is particularly relevant in Latin American contexts, where indirect costs of treatment—such as transport, loss of income, and food insecurity—can be decisive factors in treatment abandonment.

Overall, the comparative evidence suggests that Latin America does not face an isolated problem, but rather an intensified manifestation of a global challenge: treatments are interrupted when health systems fail to adapt to patients’ social realities. Therefore, reducing treatment abandonment requires preventive models capable of early risk identification, integration of social support, strengthening of community networks, use of technology with an equity-oriented approach, and adaptation of follow-up to complex life trajectories.

An important observation emerging from the comparative synthesis is that the determinants identified across Latin American countries rarely operate in isolation. Instead, they appear to accumulate and interact throughout the care trajectory. Poverty may amplify geographical barriers; geographical barriers may reduce continuity of follow-up; and weak follow-up systems may further exacerbate the effects of social vulnerability. This cumulative interaction suggests that treatment abandonment should be understood as the consequence of interconnected biopsychosocial processes rather than as the result of isolated risk factors. Consequently, interventions targeting a single determinant are unlikely to achieve sustainable improvements unless integrated within broader retention-of-care strategies.

Ultimately, retention in care should be recognised as a central indicator of programme quality. Health systems that merely record treatment abandonment without examining the underlying conditions that drive it risk inappropriately shifting responsibility onto the patient. In tuberculosis, cure depends not only on prescribing an effective pharmacological regimen, but also on sustaining the individual throughout the entire therapeutic trajectory.

These findings carry important implications for public health policy in Latin America. Tuberculosis control programmes should move beyond treatment delivery indicators and incorporate retention-focused metrics that reflect continuity of care across the care continuum. This requires the integration of social protection mechanisms, decentralised and flexible service delivery, strengthened community-based follow-up, and the use of digital tools appropriately adapted to local contexts. Policy frameworks should prioritise early risk identification and implement differentiated care pathways for high-vulnerability populations, aligning programmatic responses with the complex social realities that shape treatment adherence.

Based on the regional and comparative synthesis, a conceptual framework entitled the Latin American Integrated Model for Retention in Tuberculosis Care (MILERTB) is proposed as a hypothesis-generating model aimed at preventing treatment abandonment and loss to follow-up through an anticipatory and person-centred approach (Figure 3). The model should be interpreted as a conceptual framework to guide programmatic design, rather than as a predictive tool in its current form. It conceptualises tuberculosis care as a dynamic trajectory that begins before treatment—within the social and biological conditions that favour active disease—and extends through diagnosis, treatment initiation, follow-up, cure, and relapse prevention. At each stage, clinical, psychological, socioeconomic, and institutional determinants interact, potentially facilitating continuity or leading to disengagement from care.

Conceptually, the model is organised around three interconnected components. First, it recognises tuberculosis care as a continuum extending from exposure risk and diagnosis to treatment completion and relapse prevention. Second, it identifies the principal determinants that may facilitate or hinder retention in care throughout this trajectory. Third, it links these determinants to differentiated intervention strategies, promoting a progressive response according to the patient’s level of vulnerability and support needs.

The central operational component of the model is early risk stratification, through the initial identification of factors such as poverty, previous treatment abandonment, HIV, drug-resistant tuberculosis, substance use, deprivation of liberty, migratory mobility, geographical distance, and psychosocial vulnerability. According to the identified profile, tiered responses are activated: standard follow-up for low-risk patients; enhanced support for intermediate risk; and intensive interdisciplinary management for high-risk cases, including social support, mental health care, digital monitoring, community-based visits, and intersectoral coordination [35].

The model also incorporates differentiated care pathways for priority populations, recognising that equity does not imply uniformity. People deprived of liberty, Indigenous populations, migrants, adolescents, household child contacts, and patients with drug-resistant tuberculosis require specific retention strategies that are culturally appropriate and adapted to their real-world barriers. Such validation would enable the transition of the model from a conceptual framework to an operational tool for programme design and evaluation. Future research should evaluate the feasibility, acceptability, and effectiveness of the MILERTB framework through implementation studies, expert consensus processes, and prospective validation in diverse healthcare settings.

Finally, MILERTB redefines programme success not solely as bacteriological cure, but as the system’s capacity to sustain individuals within care throughout the entire therapeutic process. From this perspective, retention in care becomes an indicator of healthcare quality, social justice, and institutional capacity.

## 5. Limitations

This study has several limitations inherent to its narrative and interpretative design. First, no formal quality appraisal or risk-of-bias assessment was conducted, as the primary objective was conceptual integration rather than quantitative evaluation of evidence. Consequently, the findings should be interpreted as hypothesis-generating and intended to support conceptual understanding rather than establish definitive causal inferences. Furthermore, the absence of quantitative synthesis prevents estimation of the relative strength or magnitude of individual determinants associated with treatment abandonment.

This synthesis is subject to several limitations. First, the heterogeneity of the included studies—both in methodological design and in the operational definitions of treatment abandonment and loss to follow-up—may have affected comparability across contexts. In addition, variability in how these outcomes were defined and measured across studies may have further influenced the consistency of the findings.

Second, the unequal distribution of evidence among countries, with a greater concentration of studies in some settings and limited or absent data in others, restricts the generalisability of the findings at the regional level. This imbalance should be interpreted as a reflection of the current state of the evidence rather than of the true epidemiological burden.

Third, reliance on secondary data sources limits the ability to perform a standardised assessment of the quality and completeness of health information systems, potentially leading to underestimation of phenomena such as unrecorded loss to follow-up.

No formal quality appraisal tool was applied to the included studies, as the objective of the present work was conceptual integration and critical interpretation rather than systematic evidence grading. Nevertheless, study selection considered methodological relevance, thematic consistency, and contribution to the analytical framework. Similarly, no formal risk-of-bias assessment was undertaken, as the study was not designed as a systematic review. Consequently, the findings should be interpreted as a critical synthesis of recurring patterns and determinants rather than as a graded assessment of the strength of evidence.

Finally, the interpretative and narrative nature of this synthesis may have influenced study selection and evidence interpretation, as inclusion was guided by thematic relevance and conceptual contribution rather than predefined quantitative criteria. Moreover, the absence of quantitative synthesis precludes estimation of effect sizes and limits direct comparison of the magnitude of associations across studies. Consequently, the findings should be interpreted as indicative of consistent patterns and relationships rather than as evidence of causality.

Furthermore, the objective of this study was not to perform a quantitative evidence synthesis or meta-analysis, but rather to identify, organise, and critically integrate recurring determinants and patterns reported across heterogeneous Latin American and international contexts.

## 6. Conclusions

Treatment abandonment and loss to follow-up in tuberculosis in Latin America are configured as phenomena associated with the interaction of social vulnerability, clinical complexity, and health system barriers across the care trajectory. The evidence indicates that these factors do not operate in isolation, but rather accumulate and interact, shaping the care process from stages prior to treatment initiation.

From a biopsychosocial perspective, the findings suggest that, in tuberculosis, social and psychological determinants do not merely accompany the biological process but may decisively influence it throughout the entire continuum of care. These factors operate from prevention and health promotion, influence access and diagnosis, and continue to modulate adherence, retention in care, and treatment outcomes. In this context, retention in care emerges as a transversal axis of the health system response, highlighting the need to address tuberculosis through integrated strategies that consistently articulate clinical, social, and psychosocial components across all phases of the care process.

Advancing towards integrated, anticipatory, and person-centred models of care is essential to reduce treatment abandonment and strengthen tuberculosis control in the region. Reframing treatment abandonment as a systemic failure rather than an individual behaviour may redefine the future of tuberculosis control strategies.

## Figures and Tables

**Table 1:** Distribution of references included in the analytical synthesis.

Component of the Manuscript	References Included	No. of References	Purpose Within the Study
Conceptual support for the Introduction	1–4	4	Provided the global and regional context of tuberculosis, continuity of care, and social determinants of health.
Latin American studies included in the Results synthesis	5–27	23	Formed the primary evidence base for the thematic analysis of determinants associated with treatment abandonment and loss to follow-up in Latin America.
International studies included in the comparative critical reflection (Discussion)	28–40	13	Supported the comparative discussion by providing evidence on adherence interventions, social determinants, health information systems, digital technologies, and retention-in-care strategies from other world regions.
Total	1–40	40	Final corpus included in the analytical synthesis.

Source: Prepared by the authors based on the classification and analytical organisation of the studies included in this analysis.

**Table 2:** Comparative synthesis of evidence, magnitude of the problem, and implementation of strategies related to tuberculosis treatment abandonment in Latin America.

Country	Level of Evidence	Magnitude of the Problem	Implementation of Strategies	Main Problems Identified	Main Strategies Reported
Brazil	High	High	Medium	Social vulnerability, substance use, HIV coinfection, regional inequalities	Predictive models, cash transfer programmes, programme strengthening
Peru	High	High	Medium	Economic barriers, treatment fatigue, family dependency, logistical barriers	Social support, health education, digital monitoring, active follow-up
Colombia	Medium	Medium–High	Low–Medium	Poor continuity of care, administrative delays, weak programme follow-up	Limited health education, fragmented interventions
Mexico	Medium	Medium	Low	Rural dispersion, geographical barriers, delayed diagnosis, socioeconomic vulnerability	Limited access interventions, weak follow-up
Ecuador	Low	High	Low	Drug-resistant TB, prison population, treatment complexity, adverse effects	Limited programme coordination, restricted interventions
Paraguay	Low	Medium–High	Low	Economic vulnerability, prison population, Indigenous communities, limited support	Minimal documented strategies
Chile	Low	Medium	Medium	Migration, overcrowding, socioeconomic inequality	System-level responses, partial interventions
Argentina	Low	Medium	Medium	Economic barriers, outpatient adherence challenges	Community-based strategies, psychosocial support, digital reminders

Source: Prepared by the authors based on the synthesis of the included studies. Note: Levels of evidence were classified as high, medium, low, or absent based on the number and consistency of studies identified. The magnitude of the problem reflects the convergence and frequency of reported determinants associated with treatment abandonment. The implementation of strategies was categorised according to the presence and diversity of interventions described. The category “not evaluable” was reserved for contexts with absence of evidence and is not presented as a ranking but as a map of available knowledge.

## Data Availability

The data that support the findings of this study are available from the corresponding author upon reasonable request.

## Supplementary Materials

The following supporting information can be downloaded at: https://www.mdpi.com/article/10.3390/diseases14080267/s1, Table S1: Characteristics of the 23 Latin American studies included in the primary evidence synthesis.
