# Supplementary material for: Rethinking Tuberculosis Treatment Abandonment in Latin America: A Biopsychosocial Perspective and an Integrated Model for Continuity of Care
Source: Diseases. 2026 Jul 24;14(8):267. doi: 10.3390/diseases14080267 (PMC13512486; doi:10.3390/diseases14080267)
Supplement: Supplementary file 1 [file diseases-14-00267-s001.zip › diseases-4371161-supplementary.pdf]

Supplementary Materials

Supplementary Table S1. Characteristics of the 23 Latin American studies included in the primary evidence synthesis.

Author (Year)	Country	Study design	Population/Setting	Main determinants associated with abandonment or LTFU	Main findings
Lima et al. (2023)	Brazil	Retrospective cohort	TB patients	Poverty, low education, unemployment, substance use	Social vulnerability increased risk of LTFU
Ryuk et al. (2024)	Brazil	National registry analysis	259,484 TB patients	HIV, comorbidities, social vulnerability	Predictors of unsuccessful treatment outcomes identified
Lucena et al. (2023)	Brazil	Systematic review	TB patients	Poverty, weak follow-up, regional inequalities	Multiple determinants associated with treatment abandonment
Mendonça et al. (2025)	Brazil	Epidemiological study	National TB population	Socioeconomic and health service factors	Treatment interruption linked to structural and service barriers
Chenciner et al. (2021)	Brazil	Nationwide retrospective cohort	Adolescents and young adults	Social instability, poverty, vulnerability	Increased risk of unfavourable outcomes
Sánchez-Pérez et al. (2024)	Mexico	Observational study	Chiapas Highlands population	Poverty, rural dispersion, geographical barriers	Lower treatment success and continuity

Author (Year)	Country	Study design	Population/Setting	Main determinants associated with abandonment or LTFU	Main findings
Varela et al. (2023)	Colombia	Retrospective study	Previously treated TB patients	Relapse, retreatment, clinical complexity	Higher probability of unsuccessful treatment
Perlaza et al. (2023)	Colombia	Observational study	Public health network	Administrative barriers, poor follow-up	Health system weaknesses associated with abandonment
Tapia-Tenorio et al. (2023)	Peru	Cross-sectional study	TB patients	Economic barriers, employment difficulties	Increased abandonment risk
Chiang et al. (2023)	Peru	Qualitative study	Adolescents, caregivers, providers	Treatment fatigue, family dependence	Multiple barriers and facilitators of adherence identified
Brooks et al. (2024)	Peru	Prospective cohort	Child household contacts	Follow-up failures across care continuum	Significant loss to follow-up documented
Escobar et al. (2025)	Paraguay	National analysis	TB patients	Economic vulnerability, limited support	Adherence remains a major challenge
Medina et al. (2025)	Paraguay	Population study	Prison and Indigenous populations	Incarceration, structural vulnerability	TB concentrated in high-risk groups
Muñoz et al. (2024)	Chile	Ecological study	National population	Migration, overcrowding, low income	Social determinants influenced

Author (Year)	Country	Study design	Population/Setting	Main determinants associated with abandonment or LTFU	Main findings
					TB distribution
Barreto-Duarte et al. (2024)	Brazil	Nationwide study	Retreatment patients	Previous abandonment, retreatment	Poorer outcomes and recurrent discontinuity
Flores-Treviño et al. (2019)	Mexico	Clinical study	Drug-resistant TB patients	Previous treatment, diagnostic delays	Predictors of drug resistance identified
Tatés-Ortega et al. (2019)	Ecuador	Cohort study	MDR/RR-TB patients	Prolonged treatment, adverse effects	LTFU remained frequent
Romero-Sandoval et al. (2025)	Ecuador	Prison-based study	Incarcerated population	Institutional barriers, complex trajectories	Continuity of care challenges identified
Dilas et al. (2023)	Peru	Analytical study	TB patients	Social support, quality of care	Improved adherence through nursing education
Acosta et al. (2022)	Peru	Randomised controlled trial	TB patients	Electronic monitoring intervention	Improved treatment regularity
Rodrigues et al. (2024)	Brazil	Machine learning study	National registry data	Predictive risk factors for LTFU	Early identification of high-risk patients possible
Jesus et al. (2025)	Brazil	Cohort study	Vulnerable populations	Poverty and socioeconomic factors	Cash transfer programmes

Author (Year)	Country	Study design	Population/Setting	Main determinants associated with abandonment or LTFU	Main findings
					improved outcomes
Discacciati (2025)	Argentina	Narrative review	Outpatient TB care	Multiple adherence barriers	Community and psychosocial strategies recommended

Source: Prepared by the authors based on the 23 Latin American studies included in the primary narrative synthesis [5–27].
